# Gastric volvulus through Morgagni hernia and intestinal diverticulosis in an adult patient: a case report

**DOI:** 10.1186/s12893-018-0399-y

**Published:** 2018-08-29

**Authors:** Zoya Fatima Rizwan Ladiwala, Rija Sheikh, Ayesha Ahmed, Ibrahim Zahid, Amjad Siraj Memon

**Affiliations:** 1 Dow University of Health Sciences, Karachi, Pakistan; 2 Department of General Surgery, Dow University of Health Sciences, Civil Hospital Karachi, Karachi, Pakistan

**Keywords:** Diaphragmatic hernia, Morgagni hernia, Gastric volvulus, Diverticulosis, Computed tomography, Laparotomy, Gastrostomy, Gastropexy

## Abstract

**Background:**

Morgagni’s hernia (MH) is a rare type of congenital diaphragmatic hernia with limited available literature. Late presentations are infrequent and the ones complicated due to gastric volvulus are even rarer. Another uncommon association of MH is with small bowel diverticulosis. We herein discussed a case of gastric volvulus as the content of MH, and small bowel diverticulosis present in a patient concomitantly.

**Case presentation:**

A 30 year old woman, who presented with a one year history of epigastric burning and indigestion, occasionally associated with pain and vomiting. On clinical examination, no clue to the diagnosis could be ascertained. Her chest and abdominal x-ray indicated an abnormal air-fluid level at right hemithorax, which prompted a Computed Tomography (CT) scan, showing organo-axial gastric volvulus. MH with gastric volvulus was observed during laparotomy and trans-thoracic reduction of the contents was performed, along with repair of the defect. Multiple intestinal diverticuli were also found and the largest diverticulum was excised.

**Conclusions:**

Gastric volvulus through MH is a rare but potentially life-threatening condition. Non-specific symptoms like epigastric pain and vomiting can delay the diagnosis and management, however, advanced imaging techniques like CT scan can speed up this process. After the diagnosis is made, surgical repair should be attempted regardless of symptoms.

**Electronic supplementary material:**

The online version of this article (10.1186/s12893-018-0399-y) contains supplementary material, which is available to authorized users.

## Background

Morgagni’s hernia (MH) is an uncommon birth defect accounting for about 3% of all congenital diaphragmatic hernias, with substantial morbidity and mortality [1]. It is a result of abdominal contents entering the thoracic cavity through an antero-medial defect of the diaphragm and is usually diagnosed early in life [2]. MH rarely presents with gastric volvulus [3], which is the abnormal torsion of the stomach along its longitudinal or horizontal axis [4]. Another unusual incidence of MH is with small bowel diverticulosis (SBD). SBD is characterized by multiple sac like mucosal out-pouchings through weak points in the intestinal wall [5]. Here we describe a case report where gastric volvulus was herniating through the foramen of Morgagni and the patient had concurrent SBD. To the best of our knowledge such a case has never been reported previously.

## Case presentation

**Fig. 1:**
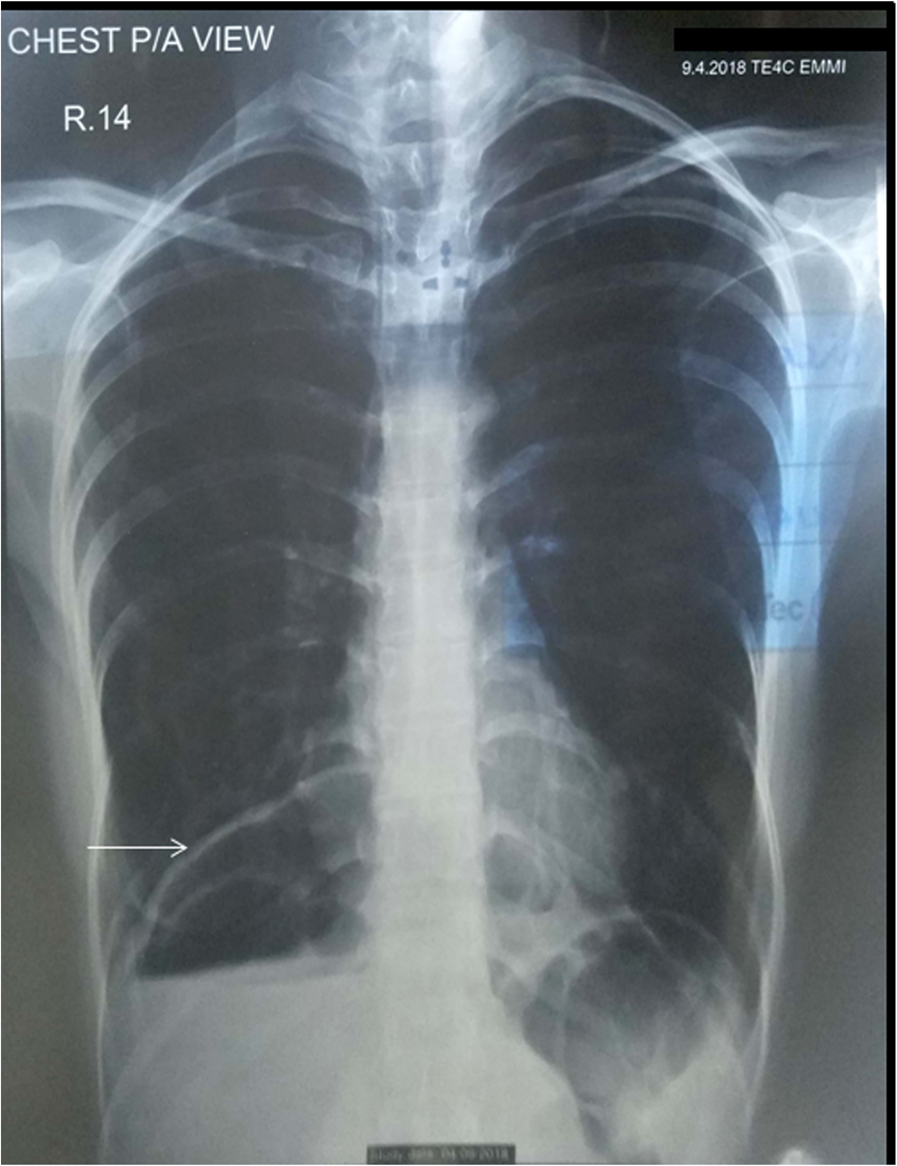
Plain postero-anterior chest X-ray showing abnormal air fluid level (white arrow) in the right basal hemi-thorax above the diaphragm

**Fig. 2:**
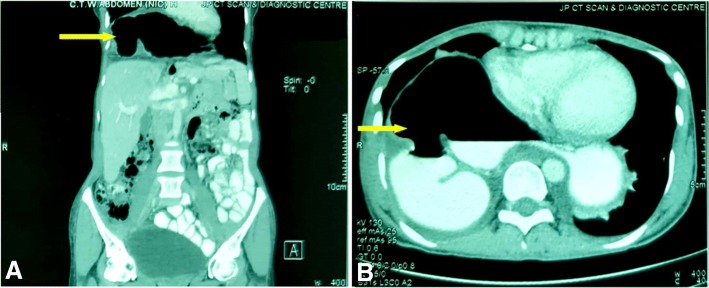
Preoperative coronal (**a**) and axial (**b**) computed tomography slice showing gastric bubble (yellow arrow) indicating herniation of stomach into the thorax

A 30-year-old female presented with complaints of epigastric burning and indigestion for 1 year, which was occasionally associated with pain and vomiting. On a previous oesophago-gastroduodenoscopy, multiple oesophageal ulcers were noted, located from 30 to 35 cm and the mucosa was seen to be circumferentially hyperaemic. Upon investigation, chest and abdominal X-ray showed abnormal air-fluid level at right hemithorax as shown in Fig. 1. Computed Tomography (CT) scan demonstrated organo-axial gastric volvulus accompanied with right hemi-diaphragm elevation and a slight mediastinal shift to the left, with gastric bubble above the diaphragm (Fig. 2), and sections through the lower chest showed mild bilateral pleural effusion with basal atelectasis of the right lower lobe. The small and large bowel loops were unremarkable and there was no evidence of bowel obstruction.

**Fig. 3:**
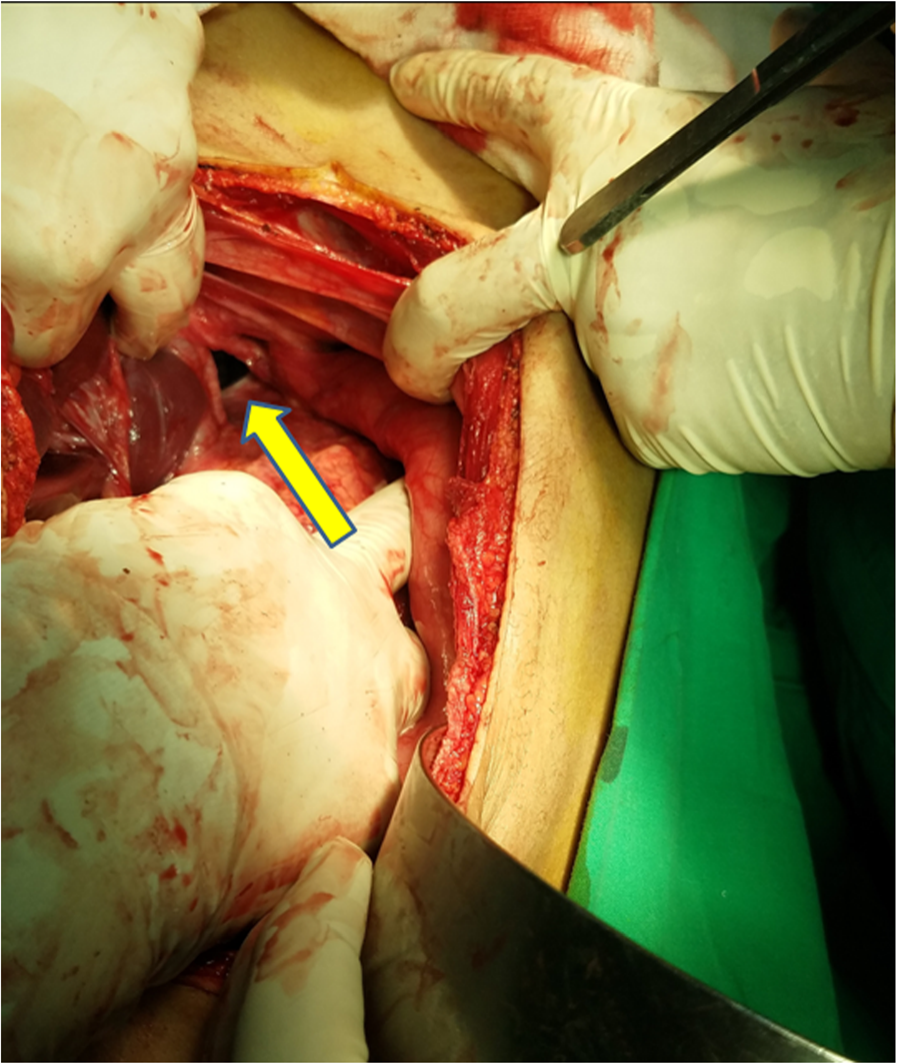
Intra-operative view of Morgagni’s defect (yellow arrow)

**Fig. 4:**
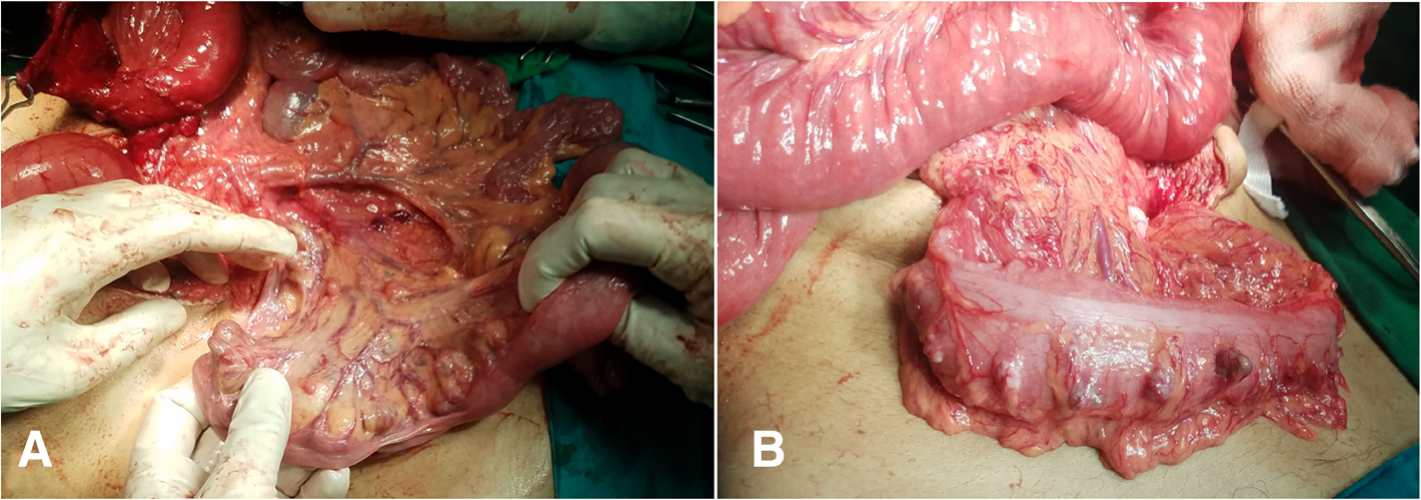
Intra-operative picture showing small bowel (**a**) and large bowel (**b**) diverticulosis

**Fig. 5:**
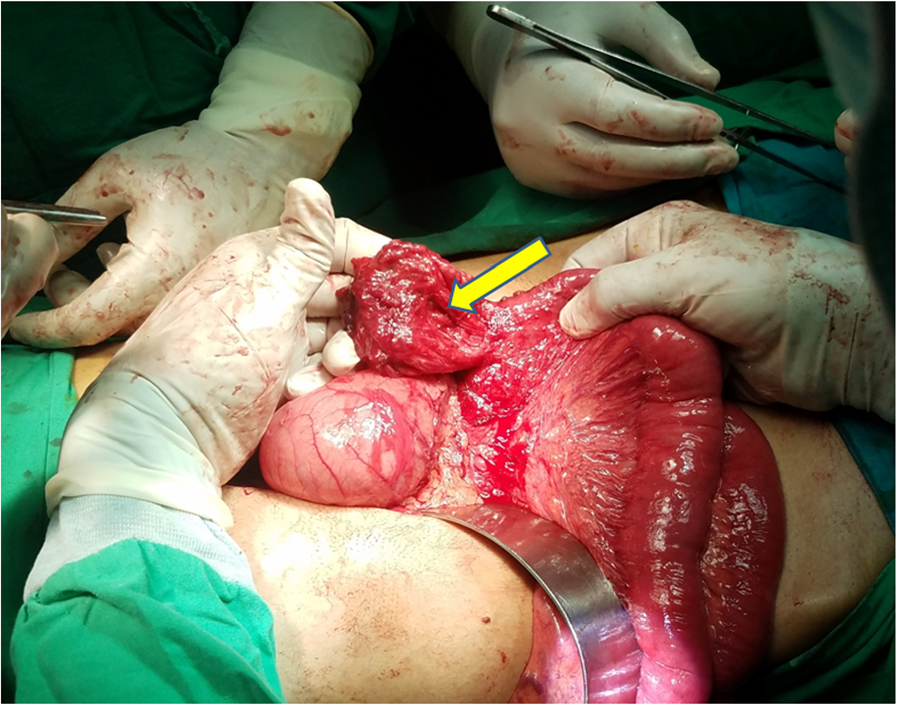
Intra-operative picture of a large jejunal diverticulum (yellow arrow)

**Fig. 6:**
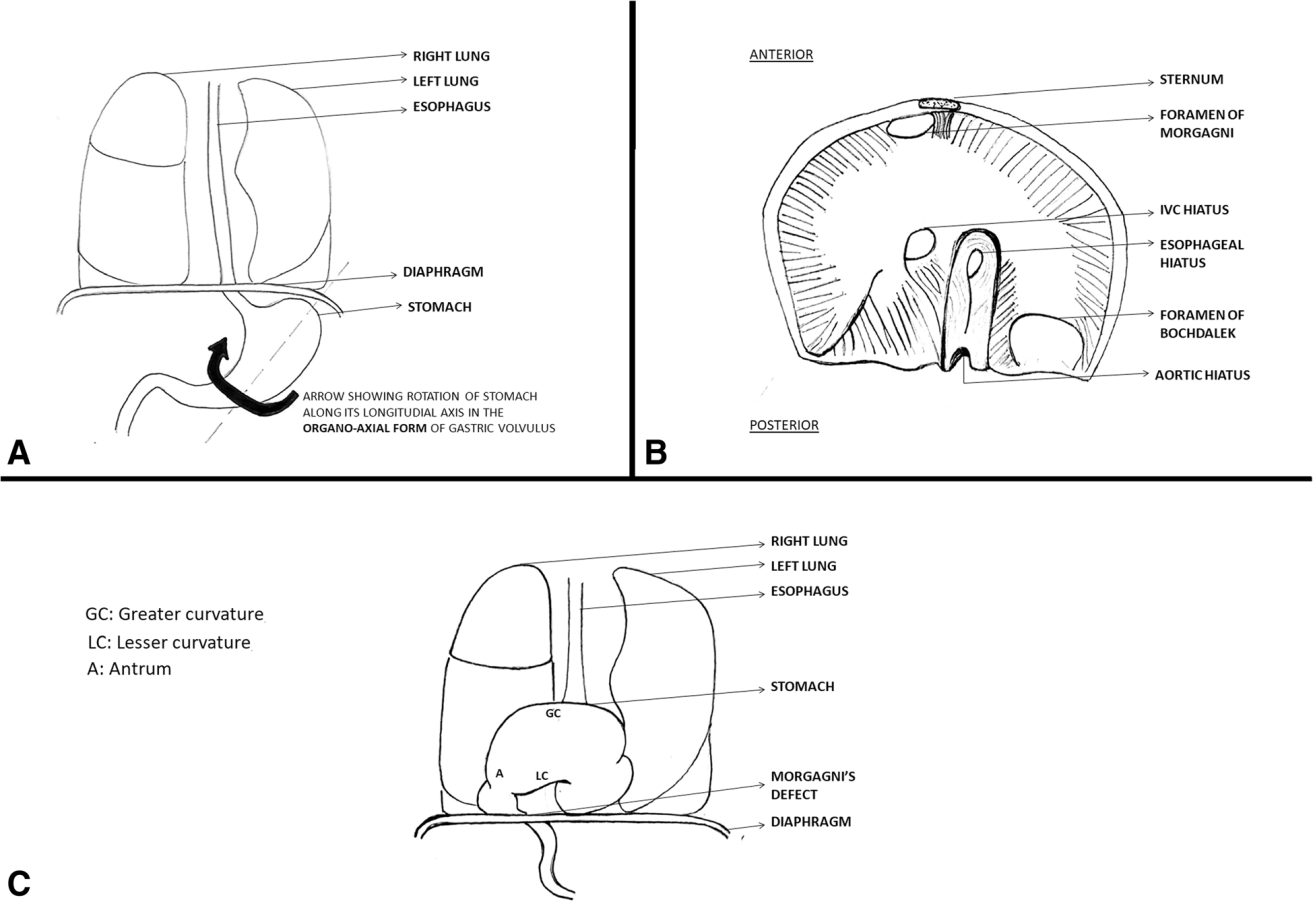
**a** Normal anatomic diagram with an arrow showing the axis of rotation of stomach in this case. **b** Axial section of the diaphragm showing the presence of foramen of Morgagni in the right antero-medial part. **c** Anatomical schema of the case showing the greater curvature of stomach being displaced superiorly, after its organo-axial rotation, and the herniating stomach into the thorax through the defect (of Morgagni) in the diaphragm

An elective laparotomy was performed through a midline incision. Stomach was not seen in the abdominal cavity, but pull on the gastrocolic ligament revealed the greater curvature of the stomach through foramen of Morgagni in the right hemi-diaphragm, with the defect measuring 4 × 5 cm (Fig. 3). The hernia was reduced, with excision of the hernial sac and the defect was repaired using a size zero non-absorbable polypropylene suture—no mesh was placed. Since there was a high jejunal repair, a gastrostomy was created which served to secure the stomach in place. Following the reduction of hernia, on further exploration, multiple diverticuli were observed in the small and large intestine (Fig. 4). Interestingly, these were unremarkable on CT scan. Only the largest and most proximal jejunal diverticulum (Fig. 5), which was about 6 cm in size, was resected using a linear stapler as it had a narrow neck. A pelvic drain was placed and the wound was closed in layers using absorbable polyglactin suture. Figure 6 exhibits the normal anatomy and a schematic diagram of this case depicting the presence of gastric volvulus through MH on the right side. The postoperative period was uneventful and the patient was discharged on the 10th post-operative day. The patient was stable and asymptomatic on follow up after one month, and she is doing well as of writing of this report. ‘Timeline for Case Report’ in Additional file 1 represents the events of this case in a chronologic order.

## Discussion and conclusions

MH is the rarest form of congenital diaphragmatic hernia which occurs through a developmental defect as a result of failure of fusion between the sternal and costal portions of the muscle [6]. Usually it occurs on the right side where it contains the omentum, followed by colon and small intestine [7]. Conversely, left-sided hernias commonly bear the stomach as reported by a review done in India [8]. Our case however, presented with a right sided hernia associated with gastric volvulus. These hernias are congenital in origin, but an acquired increase in abdominal pressures such as vomiting or coughing can cause true herniation [8]. MH may present with acute symptoms usually caused by respiratory distress or volvulus of its contents; it can also be detected incidentally with mild generic respiratory/bowel symptoms [8]. It is interesting to note that our case presented with enteric symptoms only, despite having lung abnormalities which were found on the CT scan.

Diagnosis of MH is facilitated by chest x-ray, abdominal x-rays, barium studies or ultrasound, but currently CT scan is the diagnostic modality of choice [9]. The scans must include sections through the lower chest as well as the abdomen to determine the extent of the hernia [1]. Treatment is primarily based on surgical repair [9], even if the hernia sac only contains the omentum [10], and trans-abdominal approach with interrupted non-absorbable sutures is widely preferred [9]. Nevertheless, recent advances in thoracoscopic [9] and laparoscopic [3, 9] techniques offer innovative approaches to its management [9]. In fact, laparoscopy has been concluded as the gold standard procedure for uncomplicated MH [11]. In addition to being minimally invasive, it offers better visualization of the surgical field, easy manipulation and accessibility, aesthetic benefit, fewer complications and faster recovery of the patient [10]. Several laparoscopic techniques are being practiced, which include primary closure of the defect with different variety of intra-corporeal sutures, staples or mesh [12], but no single technique has been advocated as ideal. We opted for an open surgical approach due to limitation of resources and required surgical expertise for laparoscopic repair of MH.

Literature revealed very few cases of gastric volvulus into the foramen of Morgagni, with presenting features in most cases being hematemesis, melena, abdominal bloating, and vomiting [10, 13]. Gastric volvulus occurs in conditions which increase the laxity of structures supporting the stomach, such as pyloric stenosis, pyloric hypertrophy or chronic gastric distension [10]. Several predisposing factors, such as hiatal or incisional hernias, eventration of the diaphragm, trauma, surgical injury, gastric ulcers and neoplasms, are found in about half of the patients who present with this disease [3]. Similarly in this case, the CT scan indicated diaphragmatic eventration associated with organo-axial rotation of the herniated stomach. This type of volvulus is more commonly associated with MH than its mesentro-axial counterpart [3]. The definitive treatment of gastric volvulus is gastropexy where the stomach is fixed to the diaphragm and/or anterior abdominal wall either through a laparoscopic or an open abdominal approach [4].

In order to prevent recurrences of gastric volvulus, a temporary gastrostomy may be added to act as a diversion thereby protecting the high jejunal repair and to fix the stomach to the anterior abdominal wall, serving as a gastropexy. The location of gastrostomy tube is essential, as recurrence of volvulus has been reported after gastrostomy, acting as two fixed points of the axis [14]. Literature search revealed that Bhasin endoscopically reduced a chronic gastric volvulus in 10 patients without performing any percutaneous endoscopic gastrostomy placement, but on follow up, 3 cases eventually recurred and required surgical treatment [15]. It emphasizes the significance of adding a gastrostomy tube while managing gastric volvulus. Percutaneous endoscopic gastrostomy has an added advantage for patients who have difficulties with oral intake [16] thus permitting the fixation of the stomach together with provision of enteral nutrition.

SBD occurs due to abnormal peristalsis resulting from motor dysfunction of the gut. It is more frequently confined to the proximal jejunum and distal ileum due to a wider diameter of vasa recta in these segments [5]. Clinically the disease is asymptomatic until it presents with complications which are determined by performing a diagnostic laparoscopy [5]. Complications like perforation, abscess and mechanical obstruction identified on laparoscopy, are managed by an exploratory laparotomy allowing diverticulectomy with resection of the diseased bowel and performing primary anastomosis [17].

In conclusion, MH along with gastric volvulus and small bowel diverticulosis is a rare occurrence, and may not be identified clinically; meticulous analysis of CT scan followed by surgical exploration allows definitive diagnosis. Hernia closure using non-absorbable sutures along with gastropexy and diverticulectomy yields good results with no major sequelae.

## Additional file

Additional file 1: Timeline for Case Report. A flowchart depicting the events and plans of the case with respective dates from the time of presentation up to the latest follow up. (DOCX 38 kb)

## Abbreviations

CT: Computed Tomography
MH: Morgagni Hernia
SBD: Small Bowel Diverticulosis

## References

[1] Park A, Doyle C (2014). Laparoscopic Morgagni hernia repair: how I do it. J Gastrointest Surg.

[2] Federico JA. General Thoracic Surgery. 5. Philadelphia: Lippincott Williams and Wilkins; 2000. Foramen of Morgagni hernia; pp. 647–660.

[3] Sonthalia N, Ray S, Khanra D, Saha A, Maitra S, Saha M, et al. Gastric Volvulus Through Morgagni Hernia: An Easily Overlooked Emergency. J Emerg Med. 2013 2013/06/01/;44(6):1092–1096. 10.1016/j.jemermed.2012.11.103 23602148

[4] Rodriguez-Garcia H, Wright A, Yates R (2017). Managing obstructive gastric volvulus: challenges and solutions. Open Access Surgery.

[5] Janevska D, Trajkovska M, Janevski V, Serafimoski V (2013). Small bowel diverticulosis as a cause of ileus: a case report. Pril (Makedon Akad Nauk Umet Odd Med Nauki).

[6] Court FG, Wemyss-Holden SA, Fitridge R, Maddern GJ. Unusual case of Morgagni hernia associated with malrotation. ANZ J Surg 2003 Sep;73(9):772–773. PubMed PMID: 12956800. 10.1046/j.1445-2197.2003.02745.x 12956800

[7] Ambrogi V, Forcella D, Gatti A, Vanni G, Mineo TC. Transthoracic repair of Morgagni's hernia: a 20-year experience from open to video-assisted approach. Surg Endosc 2007 Apr;21(4):587–591. PubMed PMID: 17180292. Epub 2006/12/21. eng. 10.1007/s00464-006-9017-7 17180292

[8] Abraham V, Myla Y, Verghese S, Chandran BS. Morgagni-larrey hernia- a review of 20 cases. The Indian journal of surgery 2012 Oct;74(5):391–395. PubMed PMID: 24082592. Pubmed Central PMCID: PMC3477412. Epub 2013/10/02. eng. 10.1007/s12262-012-0431-x PMC3477412 24082592

[9] Minneci PC, Deans KJ, Kim P, Mathisen DJ. Foramen of Morgagni hernia: changes in diagnosis and treatment. Ann Thorac Surg 2004 Jun;77(6):1956–1959. PubMed PMID: 15172245. Epub 2004/06/03. eng. 10.1016/j.athoracsur.2003.12.028 15172245

[10] Coulier B, Broze B. Gastric volvulus through a Morgagni hernia: multidetector computed tomography diagnosis. Emerg Radiol 2008 May;15(3):197–201. PubMed PMID: 17701234. Epub 2007/08/19. eng. 10.1007/s10140-007-0660-7 17701234

[11] Li S, Liu X, Shen Y, Wang H, Feng M, Tan L (2015). Laparoscopic repair of Morgagni hernia by artificial pericardium patch in an adult obese patient. J Thorac Dis.

[12] Shah RS, Sharma PC, Bhandarkar DS (2015). Laparoscopic repair of Morgagni's hernia: an innovative approach. J Indian Assoc Pediatr Surg.

[13] Cybulsky I, Himal HS (1985). Gastric volvulus within the foramen of Morgagni. Can Med Assoc J.

[14] Golash V (2006). Laparoscopic reduction of acute intrathoracic herniation of colon, omentum and gastric volvulus. Journal of Minimal Access Surgery.

[15] Bhasin DK (1990). Endoscopic management of chronic organoaxial volvulus of the stomach. Am J Gastro.

[16] Baudet JS, Ammengol-Miro JR, Medin C, Accarino AM, Vilaceca J, Malagelada JR (1997). Percutaneous endoscopic gastrostomy as a treatment for chronic gastric volvulus. Endoscopy.

[17] Kassahun WT, Fangmann J, Harms J, Bartels M, Hauss J. Complicated small-bowel diverticulosis: a case report and review of the literature. World J Gastroenterol 2007 Apr 21;13(15):2240–2242. PubMed PMID: 17465510. Pubmed Central PMCID: PMC4146853. Epub 2007/05/01. eng. 10.3748/wjg.v13.i15.2240 PMC4146853 17465510

