# Supplementary material for: Gastric volvulus through Morgagni hernia and intestinal diverticulosis in an adult patient: a case report
Source: BMC Surg. 2018 Aug 29;18:67. doi: 10.1186/s12893-018-0399-y (PMC6116383; doi:10.1186/s12893-018-0399-y)
Supplement: Supplementary file 1 — Timeline for Case Report. A flowchart depicting the events and plans of the case with respective dates from the time of presentation up to the latest follow up. (DOCX 38 kb) [file 12893_2018_399_MOESM1_ESM.docx]

**Timeline**

Past Medical History is not significant

A 30 year old female presented with epigastric burning and indigestion for one year which was associated with on and off pain and vomiting.

**Blood pressure:** 110/80 mm of Hg

**Pulse:** 72 bpm

Young female of average height and built. Abdomen was soft and non-tender. Gut sounds were audible.

06-04-18

OGD done on 23-08-17 which showed multiple esophageal ulcers.

CT scan done on 26-03-18 which showed gastric volvulus with diaphragmatic eventration.

Current Plan: Baseline investigations, X-ray chest and Ultrasound abdomen.

**Initial diagnosis**: Morgagni Hernia associated with gastric volvulus.

**Elective Laparotomy:** Primary surgical repair of the diaphragmatic hernia with gastrostomy and diverticulectomy.

**Final diagnosis**: Morgagni Hernia associated with gastric volvulus and intestinal diverticulosis.

**Follow up:** Patient was vitally stable post -operatively. Active tissue was reddening with puffiness of face. Wound was dry. Pain at wound site.

**Plan:** Injection Decadron and Avil.

10-04-18

11-04-18

Patient was vitally stable. Gut sounds not audible. Chest clear with NVB. Pain at wound site. Gastrostomy drain had 75ml of dark black fluid. Urine output: 400ml.

**Plan:** Nebulize the patient 6 hourly.

12-04-18

Patient was vitally stable. Gut sounds audible. Chest clear with NVB. Pain at wound site. Gastrostomy drain had 200ml of fluid. Urine output: 1200 ml.

**Plan:** Injection Sulzone, Provas, Flagyl, Tramadol and Metoclon.

13-04-18 to

14-04-18

Patient was vitally stable. Gut sounds audible. Chest clear with NVB. Pain at wound site. Gastrostomy drain had 140ml of fluid.

**Plan:** Injection Velosef, Provas, Risek and Tramadol.

15-04-18 to

17-04-18

20-04-18

**Patient was vitally stable. Gut sounds audible. Chest clear with NVB. No pain at wound site.**

**Patient was discharged with prophylactic oral antibiotics.**

Patient is asymptomatic and vitally stable on follow up after one month with no active complaints.

Patient was vitally stable. Gut sounds audible. Chest clear with NVB. No pain at wound site.

**Plan:** Injection Velosef, Risek and 5% D/W 1000 ml.
